# Capped flexosomes for prominent anti-inflammatory activity: development, optimization, and ex vivo and in vivo assessments

**DOI:** 10.1007/s13346-024-01522-z

**Published:** 2024-02-05

**Authors:** Sadek Ahmed, Diana E. Aziz, Mohamed A. Sadek, Mai Ahmed Tawfik

**Affiliations:** 1 Department of Pharmaceutics and Industrial Pharmacy, Faculty of Pharmacy, Cairo University, Kasr El-Aini, Cairo, 11562 Egypt; 2 Department of Pharmacology and Toxicology, Faculty of Pharmacy, Cairo University, Cairo, Egypt

**Keywords:** Diacerein, Flexosomes, Storage, In vivo permeation, Skin irritancy, Histopathology

## Abstract

This study aimed to formulate diacerein (DCN)-loaded flexosomes for enhanced efficacy against osteoarthritis. A 2^3^ D-optimal design was employed, investigating the impact of surfactant type (A), surfactant concentration (%w/v) (B), and oleylamine amount (mg) (C). Flexosomes were formulated using a rotary evaporator, and Design-Expert^®^ software was utilized to statistically analyze entrapment efficiency (EE%), zeta potential (ZP), poly-dispersity index (PDI), and particle size (PS) to determine the optimum formula. The selection criteria prioritized increased ZP (as absolute value) and EE%, coupled with decreased PDI and PS. Rigorous physicochemical, in vivo, and ex vivo tests were conducted to validate the safety, stability, and activity of the optimal formula. Physicochemical assessments encompassed pH measurement, transmission electron microscopy, differential scanning calorimetry, release profiles, storage effects, and Fourier transform infrared spectroscopy. In vivo tests included permeation studies, histopathology, anti-inflammatory activity, and skin irritancy, while ex vivo tests focused on permeation parameters and skin deposition. The optimum formula demonstrated high desirability (0.931), along with favorable EE% (90.93%), ZP (− 40.4 mV), particle size (188.55 nm), and sustained behavior. Notably, improved in vivo permeation (132 µm), skin deposition (193.43 µg/cm^2^), and antinociceptive activity (66%) compared to DCN suspension (48 µm, 66.31 µg/cm^2^, and 26%, respectively) were observed. The optimal formula also exhibited excellent safety and storage characteristics. In conclusion, DCN-loaded flexosomes exhibit significant potential for effectively managing osteoarthritis.

**Graphical Abstract:**

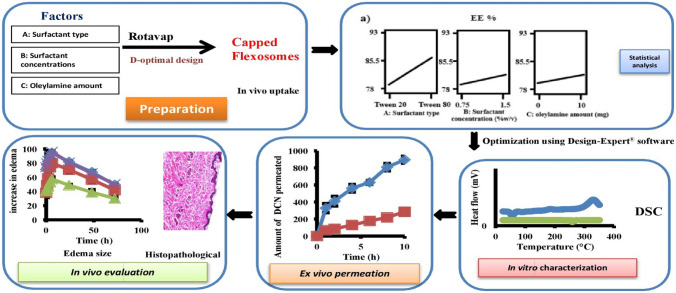

## Introduction

Skin, the largest human organ, provides a protective obstacle against injury, microbial infection, and water loss. In adults, its surface area could reach 2 m^2^ [1]. It could be anatomically divided into the epidermis (outer layer), dermis (middle), and hypodermis (inner). The epidermis is composed of many layers. The stratum corneum (SC) is the superficial part of the epidermis that mainly serves as a biological barrier against the external environment. However, the dermis contains the sebaceous glands, hair follicles, sweat glands, blood vessels, and nerve endings, thereby regulating the body temperature, skin nutrition, and sensation. Furthermore, the hypodermis is composed of adipose tissue and is responsible for energy storage, body insulation, and skin connection with bones and muscles [2].

Osteoarthritis (OA) is a degenerative joint disorder with high prevalence after 40 years. It affects the quality of life since it is a major cause of chronic pain and disability among adults [3]. About 7.6% of the global population suffered from osteoarthritis in 2020. Osteoarthritis can involve most of the joints. However, the weight-bearing joints, including the hip, knee, and hand joints, are the most affected [4]. Diacerein (DCN) is used to manage OA since it prevents cartilage degeneration through the inhibition of its causative source, namely, the interleukin-1β. Thereby, it could prevent the progression of OA and resolve its symptoms. Notably, DCN is considered SMOAD which stands for structural-modifying osteoarthritis drug [5, 6]. Unfortunately, DCN has a low aqueous solubility (3.197 mg/L) that is accompanied by poor bioavailability, short half-life (4 h), and diarrhea after oral administration, reducing patients’ compliance [7]. Therefore, transdermal delivery of DCN would be promising.

Transdermal delivery is a preferable route of administration since it provides a painless application, sustained activity, steady concentration, patient compliance, and flexible termination. Transdermal delivery faces some challenges like size restriction, irritation, and permeation. Therefore, safety and permeability tests are extremely important to verify effective application [8]. Flexosomes are recent nanosystems with enhanced flexibility. High flexibility provides added permeation and therapeutic activity. Flexosomes are composed of phospholipids, ethanol, and Tween as an edge activator. Phospholipids compose the bilayer and impart lipophilicity to the medium. However, surfactants affect drug solubility, entrapment, particle size, and stability. Furthermore, surfactants and ethanol could soften the strong connections in the stratum corneum and improve the deformability of flexosomes [9]. In order to enhance the stability, oleylamine as a capping agent was added. Capping agents could significantly prevent over-growth and maintain the physicochemical properties. Oleylamine is an amino-based amphoteric compound with high proton affinity. This affinity facilitates the interaction with other ingredients to form flexosomes [10]. Flexosomes represent an innovative approach in vesicular drug delivery, particularly for transdermal applications. Their unique composition, including an edge activator and capping agent, contributes to enhanced flexibility, stability, and improved drug delivery, making them a promising option for overcoming challenges associated with other lipid vesicles.

Precisely, our manuscript focused on constructing DCN-loaded flexosomes with pronounced transdermal activity adopting a thin-film hydration technique. To test the effects of surfactant type, surfactant concentration, and oleylamine amount on EE%, ZP, PDI, and particle size, a D-optimal design was selected. The optimum formula was subjected to various physicochemical, in vivo, and ex vivo tests to confirm its safety, stability, and activity. In vitro tests involved pH measurement, transmission electron microscopy, differential scanning calorimetry, release profile, effect of storage, and Fourier transform infrared spectroscopy. However, in vivo tests included in vivo permeation, histopathology, anti-inflammatory activity, and skin irritancy. Ex vivo tests incorporated the permeation parameters and skin deposition.

## Materials and methods

### Materials

DCN was obtained from Eva Company (Cairo, Egypt) as a gift. However, Sigma Chemical Company supplied oleylamine, soybean-phosphatidylcholine (PC), rhodamine B (RhB), dialysis membrane (14,000 Da), Tween 20, and Tween 80. El-Nasr Chemicals Company (Cairo, Egypt) provided formaldehyde, methanol, and ethanol (95%). Regarding other chemicals, they were of standard analytical quality and used directly.

### Animals

Separate caging of albino mice (20 ± 5 g) and adult Wistar rats (200 ± 50 g) in shifting cycles of dark and light at 25 ± 2 °C was considered. Animals’ nutrition was relayed on tap water and marketable food. Prior to ex vivo and in vivo tests, animals were examined to confirm healthy and intact skin. All the tests were approved by the Research Ethics Committee for experimental and clinical studies of the Faculty of Pharmacy, Cairo University (REC-FOPCU), Egypt (approval no. PI 3374), which followed the Guide for Care and Use of Laboratory Animals declared by the US National Institute of Health (NIH Publication No. 85–23, revised 2011).

### Methods

#### Experimental design

Flexosomes of DCN were formulated by adopting a D-optimal design (23). The explored factors were surfactant type (A), surfactant concentration (%w/v) (B), and oleylamine amount (mg) (C). The investigated levels were identified as − 1 and + 1 and were chosen after conducting preliminary tests. EE% (Y1), particle size (Y2), PDI (Y3), and ZP (Y4) were the studied responses that were statistically inspected using Design-Expert® software. Table 1 shows the actual values of the investigated factors, in addition to the selection criteria of the optimum formula [11].

**Table 1:** Factorial levels of studied independent variables together with measured responses and their desirability constraints

**Factor (independent variable)**	**Level**	
	** − 1**	** + 1**
A: Surfactant type B: Surfactant concentration (%w/v) C: Oleylamine amount (mg)	Tween 20 0.75 0	Tween 80 1.5 10

**Response (dependent variable)**	**Desirability constraints**	
Y1: EE % Y2: PS (nm) Y3: PDI Y4: ZP (absolute value) (mV)	Maximize Minimize Minimize Maximize	

*EE%* percent entrapment efficiency, *PDI* poly-dispersity index, *PS* particle size, *ZP* zeta potential

#### Flexosome formulation

A rotary evaporator (Rotavapor, Heidolph VV 2000, Burladingen, Germany) was used to formulate the flexosomes of DCN. Briefly, the surfactant (Tween 20 or Tween 80) at a concentration of (0.75 or 1.5%w/v), DCN (10 mg), oleylamine (0 or 10 mg), and PC (150 mg) were precisely weighed then admixed in a rounded-bottomed flask containing 10 mL of 7:3 mixtures of chloroform and methanol. The organic phase vaporized slowly for half an hour at reduced pressure at 60 °C and 120 rpm. Afterward, a 10% hydro-ethanoic solution was used to rehydrate the resulting thin film. The rehydrated mixture was left under the same condition of evaporation at normal pressure. Before storage at 4 °C, the constructed flexosomes were sonicated for half an hour at 25 °C to reduce PS [12, 13]. Table 2 reveals the composition of the formed flexosomes (F1–F10).

**Table 2:** Composition of DCN-loaded flexosomes with their measured responses (*n* = 3 ± SD)

**Formula**	**Factors**						
	**A: Surfactant type**	**B: Surfactant concentration (%w/v)**	**C: Oleylamine amount (mg)**	**Y1: EE %** **(Mean ± SD)**	**Y2: PS (nm)** **(Mean ± SD)**	**Y3: PDI** **(Mean ± SD)**	**Y4: ZP (mV)** **(Mean ± SD)**
**F1**	Tween 20	0.75	0	79.28 ± 2.11	243.30 ± 1.56	0.41 ± 0.01	− 31.15 ± 0.64
**F2**	Tween 20	0.75	0	79.93 ± 1.62	244.10 ± 1.84	0.29 ± 0.01	− 31.85 ± 0.21
**F3**	Tween 20	0.75	0	80.26 ± 2.35	285.85 ± 10.47	0.44 ± 0.07	− 32.95 ± 3.61
**F4**	Tween 20	0.75	10	81.24 ± 3.22	214.40 ± 7.00	0.36 ± 0.01	− 35.75 ± 0.35
**F5**	Tween 80	0.75	0	85.68 ± 1.25	259.70 ± 4.67	0.39 ± 0.07	− 36.55 ± 0.64
**F6**	Tween 80	0.75	10	90.89 ± 1.96	222.35 ± 16.26	0.42 ± 0.08	− 36.95 ± 0.07
**F7**	Tween 20	1.5	0	83.21 ± 1.89	242.40 ± 7.28	0.30 ± 0.01	− 34.70 ± 0.85
**F8**	Tween 20	1.5	10	83.41 ± 2.70	163.15 ± 1.06	0.44 ± 0.01	− 36.45 ± 0.35
**F9**	Tween 80	1.5	0	89.52 ± 3.16	242.25 ± 1.06	0.30 ± 0.01	− 36.65 ± 0.07
**F10**	Tween 80	1.5	10	92.08 ± 2.35	184.00 ± 0.42	0.27 ± 0.01	− 38.70 ± 1.98

*DCN* diacerein, *EE%* percent entrapment efficiency, *PDI* poly-dispersity index, *PS* particle size, *ZP* zeta potential

#### In vitro characterization of constructed flexosomes

##### Percent entrapment efficiency (EE%)

UV detector (Shimadzu, model UV-1601 PC, Kyoto, Japan) was employed to detect the unentrapped DCN (indirect determination) at λmax 258 nm. Concisely, 1 mL of the obtained formula was centrifuged for 1 h at 21,000 rpm and 4 °C (3K30, Sigma, Germany). The supernatant was diluted to 10 mL using PBS (pH 7.4). Spectrophotometric determination used the calibration curve (*n* = 3, *R*^2^ = 0.9994) and the following equation [14, 15]:

1 $$EE\;\%=\frac{(\mathrm{total\; amount\; of\; DCN}-\mathrm{ total\; amount\; of\; free\; DCN})}{\mathrm{total\; amount\; of\; DCN}}\times 100$$

The actual quantity employed indicates the total amount of DCN. However, the amount of DCN in supernatant stands for the total amount of free DCN.

##### Particle size (PS), poly-dispersity index (PDI), and zeta potential (ZP)

Certain volume of the obtained formula was diluted appropriately prior to detection employing Zetasizer (Model ZEN3600, Malvern Instruments Ltd. Worcestershire, UK). Averages of particle size, PDI, and ZP were determined at 25 °C utilizing the dynamic light scattering method [16, 17].

#### Optimization of the constructed flexosomes

The D-optimal design was chosen to examine the influence of the studied factors on the obtained responses of the constructed flexosomes utilizing Design-Expert® software. To elect the optimum formula (with the highest desirability), ZP (as absolute value) and EE% were maximized, while PDI and particle size were minimized. This election utilizes the numerical optimization approach taking into account the formula with the highest value (close to 1). The optimum formula was subjected to various physicochemical, in vivo*,* and ex vivo tests to confirm its safety, stability, and activity [18].

#### Physicochemical characterization of the optimum formula

##### Differential scanning calorimetry (DSC)

Thermal evaluation of pure DCN, oleylamine, PC, and the optimum formula was obtained using the calibrated (with indium 99.9%) calorimeter (DSC-50, Shimadzu, Japan). The optimum formula was lyophilized prior to DSC detection using (Novalyphe-NL 500 freeze-dryer, Savant Instruments, NY, USA) at reduced pressure. Afterward, 2 mg of the studied samples was sealed firmly in an aluminum pan that reached 350 °C at rate of 10 °C/min under inert N_2_ flow [15, 19].

##### Fourier transform infrared spectroscopy (FTIR)

FTIR peaks of DCN, oleylamine, PC, and the optimum formula were detected by FTIR spectrophotometer (model 22, Bruker, Coventry, UK). FTIR was used in order to detect the probable interaction between the used constituents. Furthermore, FTIR could also verify DCN entrapment inside the constructed flexosomes. Briefly, the studied samples were dehydrated and compressed into a disc of KBr. All samples were tested at 25 °C in range of 4000–500 cm^−1^ [20, 21].

##### Transmission electron microscopy (TEM)

TEM (JEOL, Tokyo, Japan) was used to visualize the morphology and the size of the optimum formula. Concisely, the optimum formula was diluted, dehydrated over copper rods coated with carbon then stained with phosphotungstic acid (2%). Imaging was done at 80 kV [22].

##### pH measurement

In order to ensure the safety of the optimum formula (no irritation), its pH was determined using the pH meter (model-3505, Jenway, Staffordshire, UK). About 5 mL of the optimum formula was poured into a small beaker (10 mL). Measurements were done at 25 °C in triplicates [23].

##### In vitro release

The bag dialysis technique was used to compare the in vitro release profile of the optimum formula and DCN suspension. Briefly, the dialysis membrane (14,000 Da) was left overnight in PBS (pH 7.4) which was used as the release medium. Afterward, the bag enclosed a volume of the optimum formula or DCN suspension containing 1.5 mg DCN. The bag was placed in an amber-bottle containing a 50-mL release medium [24]. The experiment was conducted at 37 ± 0.5 °C and 100 rpm utilizing a thermostatic shaker. Samples of 3 mL were removed in scheduled time intervals (0.5, 1, 2, 4, 6, 8 h), and DCN was detected spectrophotometrically at λmax 258 (*n* = 3, *R*^2^ = 0.9994). It is important to note that the sink condition was maintained by the instantaneous addition of fresh release medium after sample withdrawal [25].

##### Effect of storage

The optimum formula must preserve its physical properties, in addition to the measured responses (EE%, PS, ZP, in vitro release) after storage. The optimum formula was refrigerated (4–8 °C) for 3 months and then re-evaluated again [26]. Absence of aggregate formation and maintaining the overall appearance would indicate the physical stability. However, EE%, particle size, and ZP would be tested utilizing one-way ANOVA. Finally, the in vitro release profile would be detected using the formula of similarity factor “ƒ_2_” [27, 28]:

2 $${f}_{2}=50.{\text{log}}\{[1+\left(\frac{1}{n}\right){\sum }_{t=1}^{n}{\left({R}_{t}-{T}_{t}\right)}^{2}{]}^{-0.5}.100$$

The percentage of DCN released before and after the storage duration are *R*_t_ and *T*_t_, respectively. Similar profiles could be demonstrated when ƒ_2_ > 50 [23].

##### Gel preparation

After the formation of the optimum formula or DCN suspension, a certain amount of carpabol 934 (1% w/w) was added slowly with continuous stirring using a magnet to obtain a homogenous gel which was refrigerated (4–8 °C) overnight. In order to neutralize the resulted gel, triethanolamine was added [29, 30].

#### Ex vivo characterization

##### Ex vivo permeation

This test was approved by REC-FOPCU (approval no. PI 3374). The used skin was obtained from previously anesthetized recently born albino rats. The skin was then washed clearly with isopropanol to get rid of sticking fats. Afterward, deionized water was used to delicately wash the skin which was frozen (− 20 °C) in aluminum foil until use [31]. An open-ended tube was used to attach the skin that enclosed a certain volume of donor medium (the optimum formula or DCN suspension) containing 2 mg DCN. The skin was mounted in a 50-mL receptor medium of PBS (pH 7.4). Samples of 3 mL were removed in scheduled time intervals (1, 2, 4, 6, 8, 10 h), and DCN was detected spectrophotometrically at λmax 258 (*n* = 3, *R*^2^ = 0.9994). It is important to note that the sink condition was maintained by the instantaneous addition of fresh release medium after samples withdrawal. The studied parameters were cumulative DCN permeated per unit area (Q_10h_-_permeation_), flux at 10 h (J_max_), and enhancement ratio (ER) using the following equations [32, 33]:

3 $$J{_{\mathrm{max}}}=\frac{Amount\; of\; drug\; permeated}{Time\; X\; Area}$$

4 $$ER=\frac{\mathrm{J_{max} }\;of \;formulation}{{J_{\mathrm{max}}} \;of \;drug \;suspension}$$

##### Skin deposition

Following the ex vivo permeation test, skin was rinsed with normal saline to get rid of the adhering formula. Afterward, it was cut into little fragments and sonicated for half an hour in PBS (pH 7.4). The deposited DCN was examined spectrophotometrically as previously discussed [29, 34].

#### In vivo characterization

##### Skin irritancy test

As previously discussed safety, stability, and activity of the optimum formula are the main pillars to confirm the suitability of flexosomes to manage osteoarthritis. pH measurement gives an early indication of safety. However, skin irritancy and histopathological examination would provide a real indication. All rats were subjected to back shaving. Regarding the skin irritancy test, Wistar rats (200 ± 50 g) were segmented into two groups (three per group). The first group was used as a negative control (no medication), while the second group received the gel of the optimum formula. All the rats were visualized extensively for 48 h. Any sign of inflammation was recorded using the Draize scale where no erythema, very slight, well-defined, moderate, and severe erythema received (0, 1, 2, 3, 4) scores, respectively [35, 36].

##### Histopathological study

In the histopathological test, Wistar rats (200 ± 50 g) were segmented into three groups (three per group). Normal saline served as negative control (group I), while isopropanol served as positive control (group II). The optimum gel was applied on the backs of the third group. All treatment regimens were three times daily for 7 days. After the end of the test, animals were anesthetized to remove the skins which were cleaned delicately with water, subjected to serial alcohol dilutions, and stored for 24 h in blocks of beeswax at 56 °C. Furthermore, microtome (Leica Microsystems SM2400, Cambridge, UK) cut the skins which were examined under a microscope [37].

##### In vivo permeation

The third pillar was activity which was examined through in vivo permeation, anti-inflammatory, and antinociceptive activity. Regarding in vivo permeation, deep permeation facilitates the action of the applied formula. Permeation depth was detected through the fluorescent determination of rhodamine B (RhB) using helium–neon (595 nm) and argon lasers (485 nm). RhB (0.1% w/w) replaced DCN in the optimum formula or control. Confocal laser scanning microscopy (CLSM) (LSM 710; Carl Zeiss, Jena, Germany) was used to visualize RhB. Concisely, the albino mice were segmented into two groups (three per group) receiving the gel of either the aqueous solution or the optimum formula of RhB. Skins were removed after 3 h, washed with 10% ethanol, gently dried, and then visualized [38].

##### Anti-inflammatory activity

The second activity test examined the anti-inflammatory action of the optimum formula. Briefly, Wistar rats were segmented into four groups (three per group). The first and second acted as negative group (no medication) and placebo, respectively. However, the third and fourth groups received DCN gel and flexosomes gel. This test was performed according to Ammar et al.’s method where 0.1 mL of 4% H–CHO was sub-plantarly injected to induce inflammation [39]. After H–CHO injection, all the rats were kept for half an hour to fulfill the inflammatory effect of H–CHO. Medications were then applied transdermally, and edema size was determined at 0, 1, 2, 4, 6, 24, 48, and 72 h using a plethysmometer [40].

##### Antinociceptive activity

The third activity test examined the antinociceptive action of the optimum formula. Concisely, albino mice were segmented into three groups (three per group). These groups received no medication (group I), DCN gel (group II), or flexosomes gel (group III). This test was performed according to Adzu et al., where a single dose of 0.7% acetic acid (10 mL/kg) was injected intraperitoneally. In this test, the injection was 30 min after the transdermal application of the medications [41]. Afterward, the sum of hind limbs writhing within 5 to 15 min (after the acetic acid injection) was calculated. The antinociceptive activity was expressed as percent inhibition of hind limbs writhing [42].

##### Statistical analysis

D-Optimal statistical design was analyzed using Design-Expert software. All results were expressed as mean ± standard deviation (SD) after triplicate measurements. The level of significance for ANOVA analysis of the studied responses was *p* < 0.05. Analysis of two independent groups was fulfilled applying one-way ANOVA test [43].

## Results and discussions

### Analysis of D-optimal design

The effects of surfactant type (A), surfactant concentration (%w/v) (B), and oleylamine amount (mg) (C) as the investigated factors on the developed responses of flexosomes formulae were evaluated adopting D-optimal design. Table 3 reveals the preferred adequate precision (> 4) and the accepted connection between adjusted and predicated *R*^2^ of the studied responses [44, 45].

**Table 3:** Model analysis for studied responses

**Response**	**R**^**2**^	**Adjusted R**^**2**^	**Predicated R**^**2**^	**Adequate precision**	**Significant factors**
**EE %**	0.9631	0.9446	0.8739	17.351	A,B,C
**PS (nm)**	0.8496	0.7744	0.6497	8.518	B,C
**ZP (mV)**	0.8890	0.8334	0.7072	10.455	A,C

*EE%* percent entrapment efficiency, *PS* particle size, *ZP* zeta potential

#### Model analysis of EE%

**Fig. 1:**
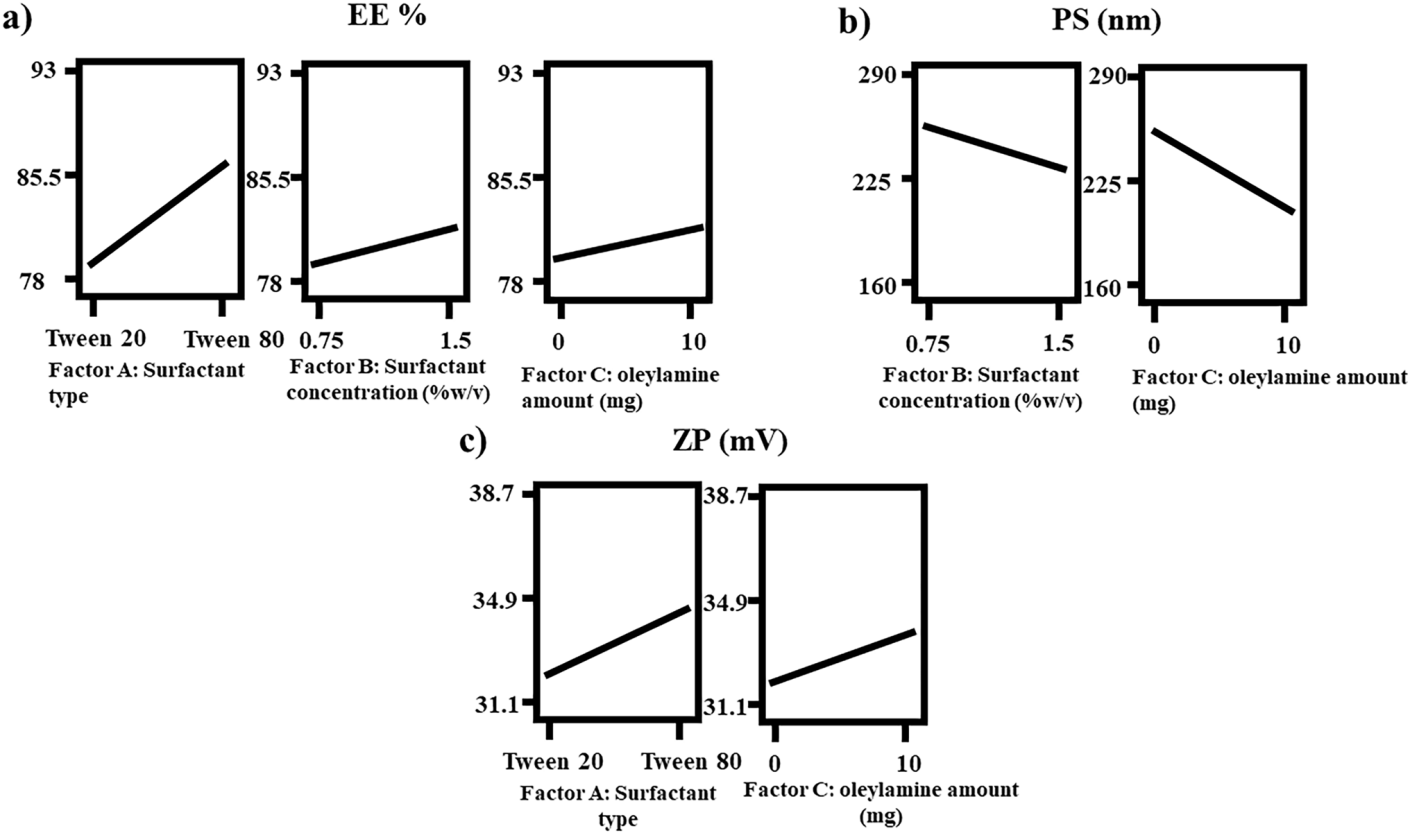
Response plots for the effect of factor A, surfactant type; factor B, surfactant concentration (%w/v); and factor C, oleylamine amount (mg) on **a** EE%, **b** PS, and **c** ZP

High EE% is crucial since the activity is directly related to delivered concentration. EE% fluctuated between 79.28 ± 2.11 and 92.08 ± 2.35%, as revealed in Table 2. ANOVA analysis found that factor A (surfactant type), factor B (surfactant concentration (%w/v)), and factor C (oleylamine amount (mg)) had a positive significant effect (*p* < 0.05). Figure 1a illuminates the effects of all variables. Equation of the coded factors was:

$$\mathrm{EE\; \% }= 85.78 + 3.76\mathrm{A }+ 1.27\mathrm{B }+ 1.12\mathrm{ C}$$

Surfactants are balanced amphiphilic compounds that have the ability to dissolve both hydrophilic and lipophilic compounds. DCN is a water-insoluble compound; thereby, surfactants will arrange themselves that polar groups will be in contact with the surrounding medium while the non-polar groups will be out of contact with the surrounding medium enclosing DCN. Incorporation of DCN inside the lipophilic core would enhance its solubility and entrapment. Tweens are ester-ether linked surfactants that are produced by reacting ethylene oxide with sorbitan fatty acid esters. The resulted Tween depends on the length of the interacting fatty acid. The longer the fatty acid (more carbons), the more lipophilic the surfactant, the lower HLB and the higher the solubility of lipophilic compounds [46]. Tween 20 (HLB = 16.7) is a polyoxyethylene sorbitan mono-laurate, while Tween 80 (HLB = 15) is a polyoxyethylene sorbitan mono-oleate. The chemical structures of lauric acid and oleic acid are C_12_H_24_O_2_ and C_18_H_34_O_2_, respectively. It is clear that tween 80 has longer fatty acid chain (more carbon) than Tween 20; thereby, more lipophilic and could entrap more DCN [47]. Moreover, increasing the amount of surfactants would produce more lipophilic bilayer shelter that entrap DCN; thereby, type and amount of surfactant significantly (*p* < 0.05) affect EE%.

Oleylamine is a strong capping agent that has long-chain fatty acid (C_18_H_37_N) that imparts an enhanced flexosomes lipophilicity and DCN entrapment. Moreover, it could produce 3D fibrillar assemblies resulted from the various non-covalent interactions (H-bonding, Van der Waals, electrostatic) that impart its gelling properties [48]. It is important to note that these fibrillary structures could significantly entrap DCN and stabilize the medium [49]. Other ingredients also augment the entrapment of DCN. Ethanol could increase the solubilization of DCN through formation of extensive H-bonds with the –COOH and C = O moiety of DCN [13, 50]. PC increases the lipophilicity of the medium that is favorable to DCN. Furthermore, rigidity of flexosomes increases due to strong interaction between ethanol and PC (H-bonding), thereby coherent layers enclosing the vesicles in addition to the increased viscosity [51, 52].

#### Model analysis of particle size

Deep permeation is facilitated by small particle size, hence affecting the activity [53]. PS varied between 163.15 ± 1.06 and 285.85 ± 10.47 nm, as revealed in Table 2. ANOVA analysis revealed that factor B (surfactant concentration (%w/v)) and factor C (oleylamine amount (mg)) had a negative significant effect (*p* < 0.05). However, factor A (surfactant type) had a non-significant effect (*p* > 0.05). Figure 1b illuminates the effects of all variables. Equation of the coded factors was:

$$\mathrm{particle \;size}=222.72 + 4.36{\text{A}}- 14.77{\text{B}}- 26.74{\text{C}}$$

Surfactants have the ability to reduce the interfacial tension (I.T) between flexosomes and adjacent medium. I.T gives an indication of the structure similarity. Lower I.T means existence of structure similarity between flexosomes and adjacent medium, thereby aggregation retards and PS decreases [54]. Furthermore, increasing the amount of surfactants would prevent the core swelling since numerous flexosomes would enclose DCN. Comparable results were previously obtained by Eldeep et al*.* working on enhancing the ocular delivery of brimonidine cubosomes and Albash et al*.* working on enhancing the topical delivery of PEGylated cerosomes of fenticonazole nitrate [55, 56]. Regarding oleylamine, the bulk structure and the capping properties control the size of the formed vesicles. The long alkyl chain of oleylamine imparts a steric hindrance effect. Moreover, surface adsorption prevents the over-growth and the interaction between adjacent vesicles, thereby PS decreases [57].

#### Model analysis of PDI

The homogeneity of the system could be detected by PDI. As PDI comes close to zero, the homogeneity of the system increases [58]. PDI fluctuated between 0.27 ± 0.01 and 0.44 ± 0.07 as revealed in Table 2. None of the studied factors revealed a significant effect against PDI (*p* > 0.05).

#### Model analysis of ZP

Presence of a sufficient charge (around ± 30) on the surface of the particles ensures effective repulsive forces that hinder the aggregation and promote the stability of the system [59]. ZP varied from − 31.15 ± 0.64 to − 38.70 ± 1.98 as revealed in Table 2. Therefore, sufficient charges were detected on the surface of all the studied formulae. ANOVA analysis confirmed that factor A (surfactant type) and factor C (oleylamine amount (mg)) had a positive significant effect (*p* < 0.05). However, factor B (surfactant concentration (%w/v)) had non-significant effect (*p* > 0.05). Figure 1c illuminates the effects of all variables. Equation of the coded factors was:

$$\mathrm{ZP }= 35.83 + 1.38\mathrm{A }+ 0.79\mathrm{B }+ 1.13\mathrm{ C}$$

Aggregation of the vesicles is retarded by high ZP (inverse relation). ZP significantly affects the stability since presence of sufficient charges on the surface of the particles produce pronounced repulsive forces that hinder over-growth and aggregation [55]. Therefore, presence of ionizable groups highly affect ZP. Among the constituents of flexosomes, ethanol and PC could impart a negative charge due to the presence of –OH group in ethanol and phosphatidyl group in PC. PC arranges the choline group inward, thereby augmenting the negative charge on the surface [13]. Tweens are non-ionic surfactants that reduce the I.T with the surrounding medium thereby preventing the aggregation. Tween 80 (C18) is larger than Tween 20 (C12), hence imparting more steric hindrance effect. Oleylamine could stabilize the interface between flexosomes and adjacent medium due to its high proton affinity and surface adsorption behavior [10, 14].

### Confirmation of the optimization process

Numerical optimization reveals the composition of the optimum formula (surfactant type = tween 80, surfactant concentration = 1.47%w/v and oleylamine amount = 10 mg with desirability = 0.931). The optimum formula revealed EE% (90.93%), particle size (188.55 nm), and ZP (− 40.40 mV). All these results were in harmony with the predicated responses confirming the optimization process (small % deviation), as shown in Table 4 [43, 60]. The optimum formula was subjected to further in vitro, ex vivo*,* and in vivo tests.

**Table 4:** Characterization of the optimum formula

**Response**	**Y1**	**Y2**	**Y4**
	**EE %**	**PS (nm)**	**ZP (mV)**
**Observed value**	90.93	188.55	− 40.40
**Predicated value**	91.94	185.56	− 39.14
**% Deviation (absolute)**	1.10	1.61	3.

*EE%* percent entrapment efficiency, *PS* particle size, *ZP* zeta potential

### Physicochemical Characterization

#### Differential scanning calorimetry (DSC)

**Fig. 2:**
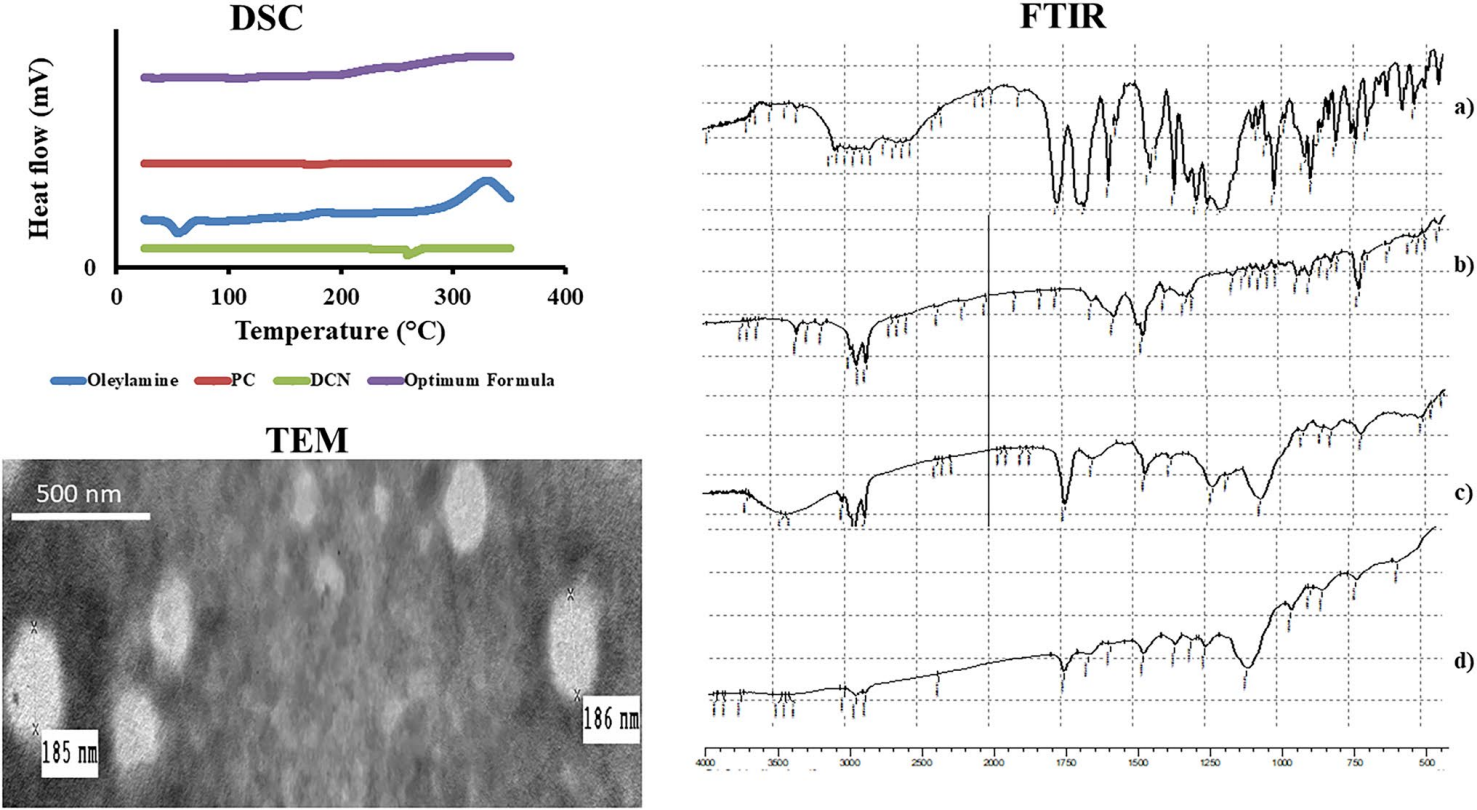
DSC thermogram and FTIR spectra of **a** pure DCN, **b** oleylamine, **c** PC, and **d** lyophilized optimum formula, in addition to TEM of the optimum formula

The thermal profiles of DCN, oleylamine, PC and the optimum formula are shown in Fig. 2**.** DCN displayed a peak around 256 °C related to its crystalline nature [15]. Oleylamine displayed a peak around 54.59 °C [14]. Moreover, PC displayed an endothermic peak around 165 °C. The thermogram of the optimum formula did not show the distinctive peak of DCN, hence confirming its complete encapsulation that is essential for effective transdermal delivery [61].

#### Fourier transforms infrared spectroscopy (FTIR)

FTIR graphs of DCN, oleylamine, PC and the optimum formula are shown in Fig. 2**.** Regarding DCN, the m-substituted benzene, –COOH, and C = O groups revealed peaks at 760, 3300, and 1764 cm^−1^, respectively [62]. Regarding oleylamine, peaks at 2860.79 and 3332.99 cm^−1^ related to C–H and N–H stretching, respectively [63]. Moreover, PC revealed peaks at 2900, 1740, 1465, and 1069 related to aliphatic C–H, C = O, C = C, and –O– bonds, respectively [36]. It is important to note that oleylamine has a strong capping properties that augments the stability of the optimum formula. Oleylamine has an amino group that could interact with the carboxylic group of DCN. This interaction was manifested through appearance of amide peaks in the FTIR graph of the optimum formula. The optimum formula revealed the amide A peak around 3330 cm^−1^ (N–H stretching) and the amide I peak around 1710 (C = O) [64, 65]. FTIR also confirmed the complete entrapment of DCN due to the disappearance of its distinctive bands. FTIR supports the previously obtained results of DSC [66].

#### TEM microscopy

As shown as Fig. 2, the obtained size verifies the results of Zetasizer. TEM also confirms the almost smooth surface round-shape particles. The capping properties of oleylamine hinder the aggregation of vesicles or formation of cluster; thereby, stable flexosomes is predicated. Moreover, the high negative charge on the surface of flexosomes (ZP =  − 40.40 mV) resulted from the ionizable groups of PC (phosphatidyl groups) and ethanol (hydroxyl). Furthermore, the surface properties of tween augment the capping effect of oleylamine [14, 67].

#### pH measurement

Early assessment of safety was confirmed through pH measurement (6.39 ± 0.15). We could expect that the optimum formula will not impart any harmful effects following the application [62]. Safety of the optimum formula would be subjected to further in vivo tests.

#### In vitro release

**Fig. 3:**
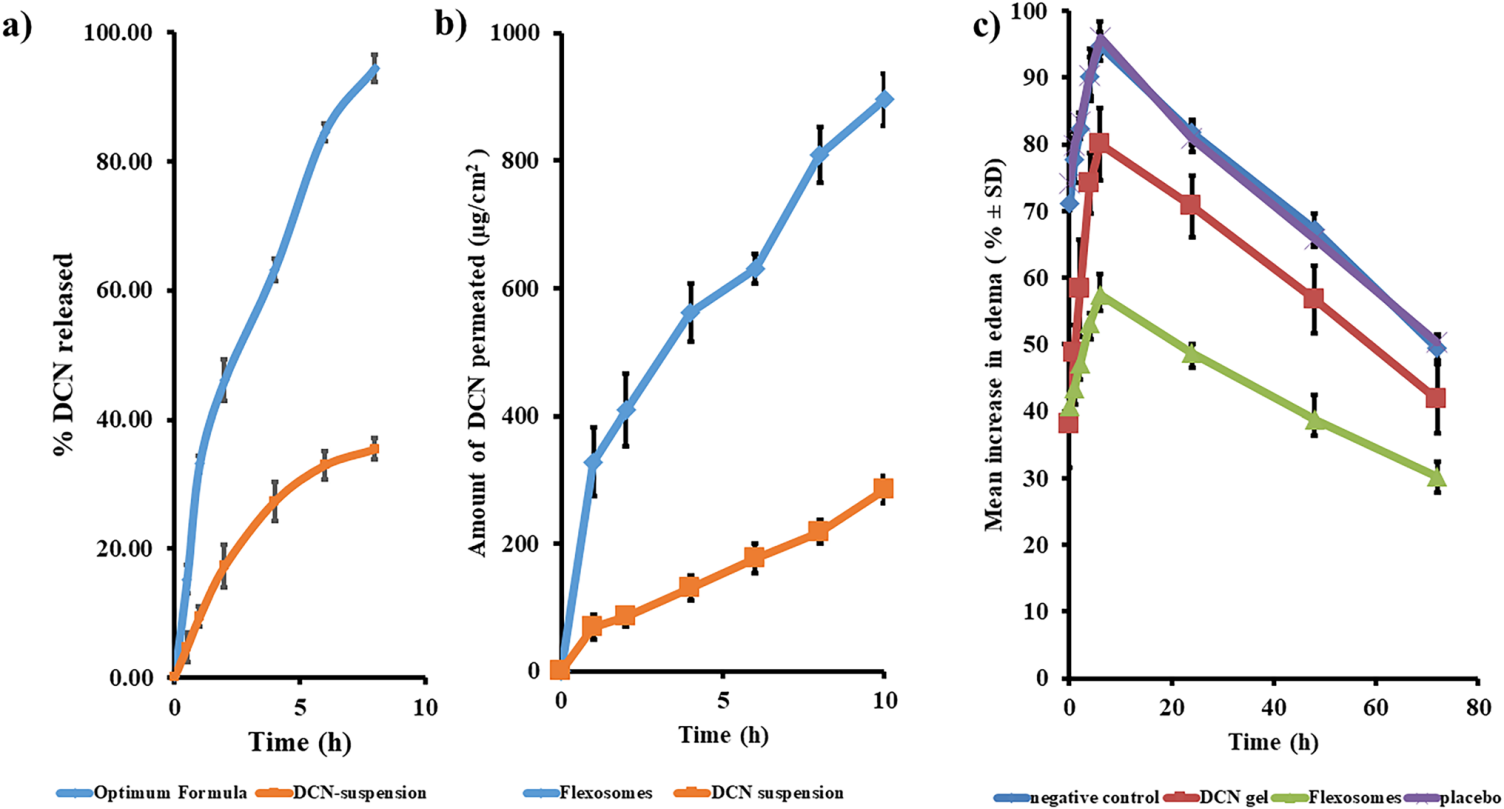
In vitro release (**a**), *ex-vivo* permeation (**b**), and anti-inflammatory activity (**c**) from the optimum formula compared to that from DCN suspension, mean ± SD, *n* = 3

The in vitro release profile of the optimum formula confirmed its enhanced activity compared to DCN suspension, as shown in Fig. 3a. Sustained activity facilitates patient compliance as a result of reducing the dose frequency. It is also clear that significantly higher (*p* < 0.05) release profile was obtained from the optimum formula. Higher and sustained release profile developed due to the unique structure of capped flexosomes. Capping effect of oleylamine significantly reduces the I.T between the vesicles and the release medium, thereby wettability and release increases [14]. Furthermore, Tween 80 facilitates the solubilization of DCN inside the hydrophobic core of the lipid bilayer and prevents the aggregation of particles, thereby reducing particle size and increasing release [68]. Ethanol could form H-bonds with the –COOH and C = O groups of DCN, thereby augmenting the solubility and release. Sustained behavior resulted from the ability of ethanol to form H-bonds with PC and tween leading to formation of rigid flexosomes [13, 50]. PC supports the sustained activity through increasing the viscosity and lipophilicity and reducing the fluidity of the medium [52]. The release profile of capped flexosomes could be segmented into rapid phase (first 2 h) followed by the sustained phase. The rapid phase resulted from release of surface unentrapped DCN; however, the sustained phase resulted from the slow release of entrapped DCN [69].

#### Stability study

The optimum formula was re-assessed after the storage duration in terms of physical properties, measured responses (particle size, EE%, and ZP) and in vitro release. Regarding the physical properties, presence of aggregates was not detected. Moreover, the optimum formula retained its previously measured responses (*p* > 0.05), as shown in Table 5. Furthermore, similar release profile after the storage period was demonstrated (ƒ_2_ = 74.22) [28, 70]. Ionization of ethanol and PC imparts a high negative charge (ZP =  − 40.40 mV); thereby, particles would have sufficient repulsive forces that prevent their aggregation. The effect of the negative charge is augmented by the small particle size, since there is an inverse relation between the particle size and surface area. The larger the surface area, the more the exposed negative charge; thereby, repulsive forces manifest [71]. Furthermore, oleylamine (capping agent) and Tween would be adsorbed on the surface of the particles preventing the over-growth and stabilizing the interface with the surrounding medium [10].

**Table 5:** Effect of short-term stability on the optimum formula

**Parameter**	**Fresh**	**Storage for** **3 months at 4–8 ºC**	
		**Value**	Probability (*p*)*
**EE %**	90.93 ± 1.45	89.52 ± 2.37	0.551
**PS**	188.55 ± 5.73	193.75 ± 4.45	0.417
**ZP**	-40.40 ± 4.53	− 44.35 ± 4.17	0

*EE%* percent entrapment efficiency, *PDI* poly-dispersity index, *PS* particle size, *ZP* zeta potential

^*^One-way ANOVA analysis to compare between the freshly prepared and the stored optimum formula

### Ex vivo characterization of the optimum formula

#### Ex vivo permeation

The optimum formula demonstrated significantly (*p* < 0.05) larger permeation profile (Fig. 3b), augmented flux (*J*_max_), and enhanced enhancement ratio (ER) (895.73 ± 41.03 µg/cm^2^, 89.57 ± 4.10 µg/cm^2^/h, and 3.14, respectively) compared to DCN suspension (285 ± 21.25 µg/cm^2^, 28.52 ± 2.12 µg/cm^2^/h, and 1, respectively). The augmented permeation properties resulted from the surface properties of Tween and oleylamine that reduces significantly the particle size and increases ZP enhancing the permeation and prolonging the retention time [43, 72]. Furthermore, surfactant and ethanol would soften the strong connections in the stratum corneum (SC) [9]. Moreover, the deformability of flexosomes through skin layer would be enhanced by ethanol [12]. These results augmented the formerly discussed in vitro release properties.

#### Skin deposition

Flexosomes showed significantly (*p* < 0.05) higher skin deposition properties (196.89 ± 14.65 µg/cm^2^) compared to DCN suspension (66.32 ± 5.86 µg/cm^2^); thereby, depot activity was confirmed [73]. Factors that supported the depot behavior were the pronounced negative charge (interaction with the cationic skin proteins), small particle size, and enhanced deformability (permeation through interstices of SC) [74, 75].

### In vivo characterization of the optimum formula

#### Skin irritancy test

Safety of the optimum formula would be subjected to further in vivo testing to confirm the aforementioned pH measurement. Regarding skin irritancy, Draize score of zero was concluded after 48 h (no erythema). Therefore, we could conclude that flexosomes are suitable for transdermal application.

#### Anti-inflammatory activity

The second activity test was anti-inflammatory behavior. After monitoring the change in edema size, it was clear that 6 h was sufficient to achieve the maximum edema which was then decreased gradually until 72 h (Fig. 3c**)**. Mean increase in edema recorded (94.71% and 49.38%) after 6 h and 72 h, respectively, for the negative control. Placebo recorded comparable results to the negative control (95.96% and 50.40%) confirming that other ingredients did not impart anti-inflammatory activity. DCN gel recorded (80.04% and 41.88%), while flexosomes recorded significantly (*p* < 0.05) lower edema size (57.41% and 30.18%). The least AUC was obtained from flexosomes; thereby, augmented anti-inflammatory activity was verified. Deep permeation, minute particle size, and augmented EE% facilitate delivering marked DCN through the skin layers.

#### Histopathological study

**Fig. 4:**
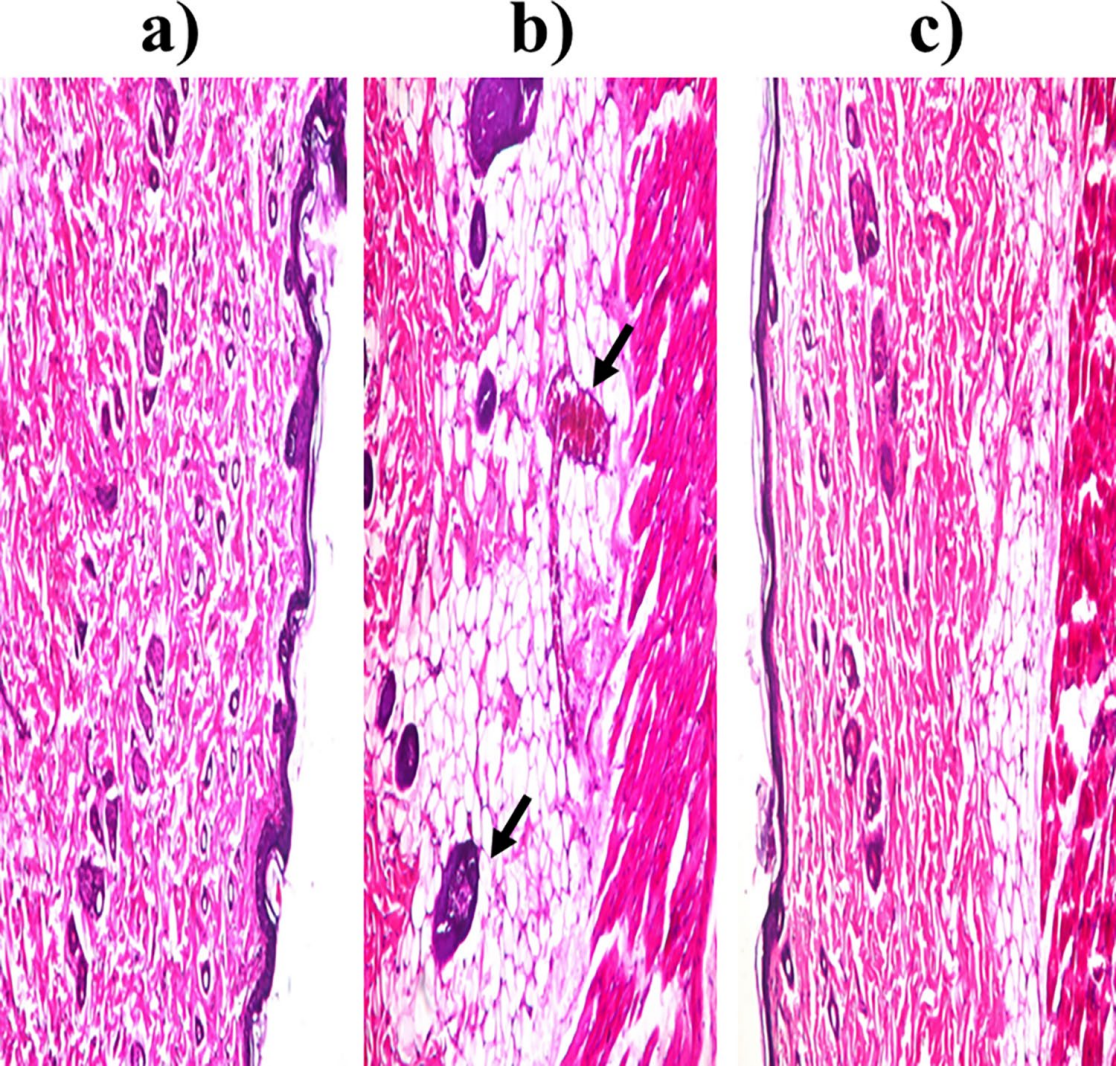
Photomicrographs of rats’ skin after instillation of **a** normal saline (negative control), **b** isopropanol (positive control), and **c** optimum flexosomes gel, *n* = 3

Figure 4 revealed the histopathological examination of negative control (a), positive control (b), and optimum formula (c). Healthy epidermis, dermis, sebaceous glands, and hair follicles were demonstrated after the application of the negative control or the optimum formula. However, the positive control demonstrated blood vessels congestion, dermis hyalinization, and epidermis focal acanthosis. So, safe application of flexosomes was augmented [15].

#### In vivo permeation

**Fig. 5:**
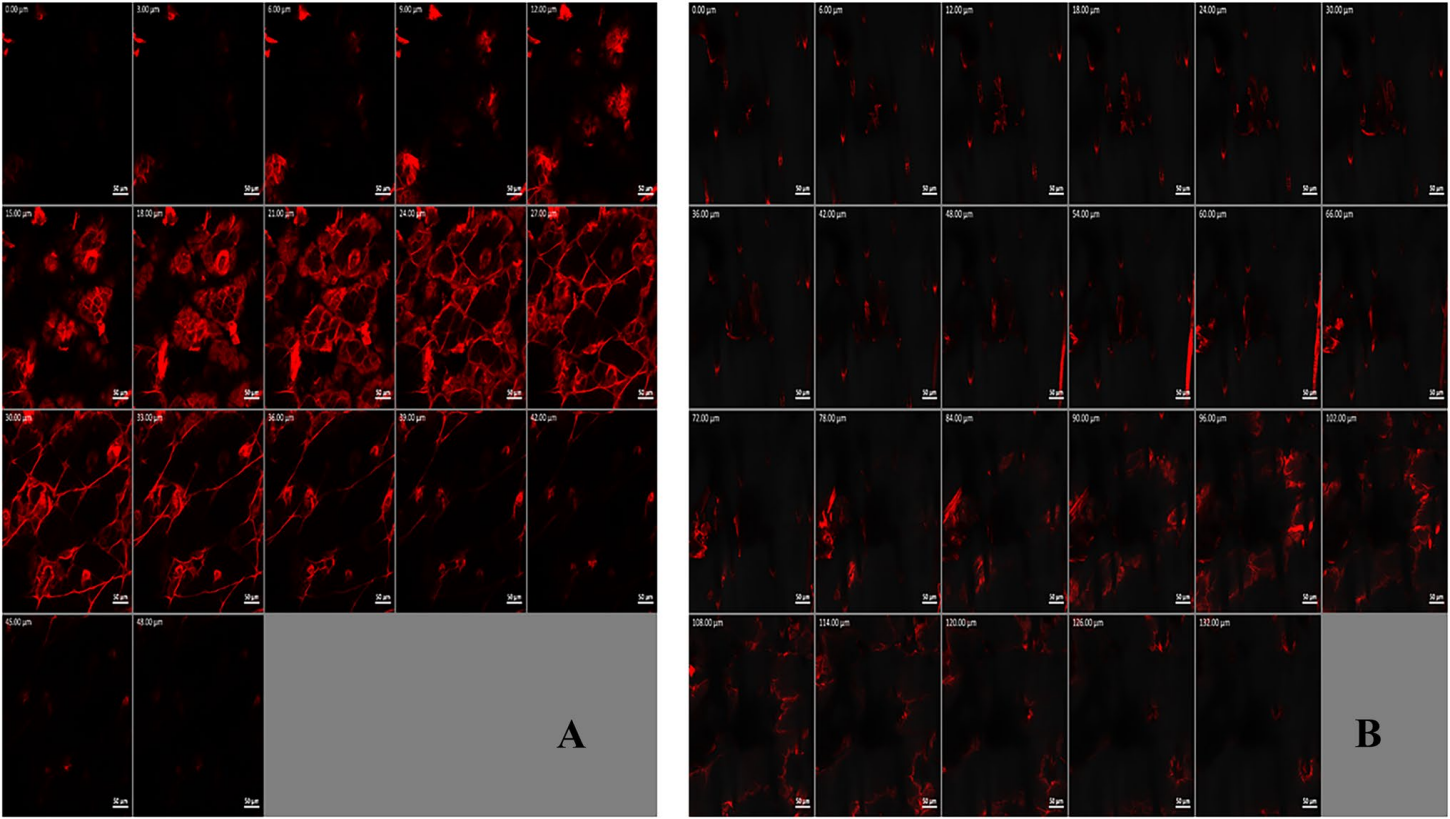
Confocal laser scanning micrographs of RhB gel (**A**) and RhB flexosome gel (**B**) *n* = 3

The third pillar for testing effective transdermal application was the activity. The first in vivo activity test examined the permeation behavior to augment the previously stated ex vivo test. As shown in Fig. 5, flexosomes revealed higher permeation of RhB compared to the RhB gel (132 and 48 μm, respectively) verifying the ex vivo test. The deep permeation resulted from small PS, oleylamine capping properties, flexosomes deformability, and SC softening effect.

#### Antinociceptive activity

The last activity test was the antinociceptive properties, recorded in Table 6. The number of writhes reduced significantly (*p* < 0.05) after the application of flexosomes compared to DCN gel. We conclude that in vivo permeation, anti-inflammatory, and antinociceptive activity gave comparable results that verified the superior activity of flexosomes. Therefore, formulating DCN as flexosomes could significantly improve the management of osteoarthritis.

**Table 6:** Antinociceptive activity of the studied formulae

**Preparation**	**Number of writhes**	**Percent inhibition (%)**
**Negative control**	72.50 ± 2.12	-
**DCN gel**	53.50 ± 3.54	26%
**Flexosome gel**	24.50 ± 2.12	66%

## Conclusions

Flexosomes of DCN were successfully prepared implementing the thin-film hydration technique. The formula with the highest desirability (0.931) was selected as the optimum formula. This formula recorded augmented EE% (90.93 ± 1.45%) that was verified through DSC and FTIR. Furthermore, minute particle size (188.55 ± 5.73 nm), accepted ZP (− 40.40 ± 4.53 mV), sustained in vitro release, high stability, homogenous dispersion, and round-shaped particles were manifested. Flexosome safety was confirmed through pH measurement (6.39 ± 0.15), Draize score (0), and histopathological examination. Flexosome activity was demonstrated through ex vivo permeation (895.73 µg/cm^2^), in vivo uptake (132 µm), antinociceptive activity (% inhibition = 66%), and anti-inflammatory activity (mean increase in edema after 72 h = 30.18%) compared to DCN suspension (285.15 µg/cm^2^, 48 µm, 26%, and 41.88%). Finally, flexosomes could significantly improve the transdermal management of osteoarthritis.

## Data Availability

All data generated or analyzed during this study are included in this published article.
